# Newcastle Disease Virus-Vectored African Swine Fever Virus Antigen Cocktail Delays the Onset of ASFV-SY18 but Is Not Protective

**DOI:** 10.3390/microorganisms12122590

**Published:** 2024-12-13

**Authors:** Qian Li, Yiqian Jiang, Min Zheng, Xuefei Sun, Lili Hui, Yanyan Zhang, Huixian Yue, Yu Qi, Siqi Li, Junnan Ke, Qixuan Li, Boli Ma, Xiaoying Jia, Fengjie Wang, Lijuan Mi, Shoufeng Zhang, Faming Miao, Shuchao Wang, Fei Zhang, Teng Chen, Rongliang Hu

**Affiliations:** Key Laboratory of Prevention & Control for African Swine Fever and Other Major Pig Diseases, Ministry of Agriculture and Rural Affairs, Changchun Veterinary Research Institute, Chinese Academy of Agricultural Sciences, Changchun 130122, China; liqian2714@yeah.net (Q.L.); 13844173048@163.com (Y.J.); zhengmin916@163.com (M.Z.); sunxuefei0322@163.com (X.S.); huilili202103@163.com (L.H.); 18843184112@163.com (Y.Z.); 18380384852@163.com (H.Y.); qiyu0204@163.com (Y.Q.); lisiqi202411@163.com (S.L.); kejunnan0125@163.com (J.K.); li.qi.xuan@foxmail.com (Q.L.); 15695013087@163.com (B.M.); 18043621937@163.com (X.J.); 15144262507@163.com (F.W.); mlj84321@163.com (L.M.); zhangshoufeng@hotmail.com (S.Z.); miaofaming81@163.com (F.M.); wsc1026@126.com (S.W.); fei2333@163.com (F.Z.)

**Keywords:** African swine fever virus, humoral and cellular immune response, cytokine, vector vaccine, Newcastle disease virus

## Abstract

African Swine Fever (ASF) is a highly contagious viral disease threatening the global pig industry. Currently, only two gene-deleted live attenuated vaccines are approved, exclusively in Vietnam, and their long-term effectiveness and safety are unproven, prompting the need for safer alternatives. This study assessed a cocktail of African Swine Fever Virus (ASFV) antigens delivered via a recombinant Newcastle Disease Virus (rNDV) vector against the genotype II ASFV-SY18. Antigens pB602L, pEP84R, and p22 (pKP177R) were selected based on virus neutralization and lymphocyte proliferation assays in mice and combined with capsid protein p72 (pB646L) for vaccination and challenge in pigs. The antigen cocktail delayed ASF symptoms by 3–4 days but did not prevent the lethal ASFV-SY18 infection. Significant ASFV-specific gamma interferon (IFN-γ) positive responses and NDV antibodies were detected post-inoculation, showing an induced immune response, though ASFV-specific p72 antibodies were absent. The cocktail did not cause cytokine imbalance, indicating the vector’s safety in pigs. Despite some delay in disease progression, the protection against genotype II ASFV was inadequate, underscoring the need to select more effective antigens and enhance immune responses for virus-vectored vaccines.

## 1. Introduction

African Swine Fever (ASF) is an acute, highly contagious viral disease that poses a significant threat to the global swine industry. First identified in Africa in the early 20th century, ASF has caused a major economic impact on pig health, production, and trade due to its high morbidity and mortality rates. The African Swine Fever Virus (ASFV), a large, double-stranded DNA virus from the *Asfarviridae* family, is the causative agent of ASF. Its structure is complex, with multiple layers including the nucleoid, core shell, inner envelope, outer envelope, and capsid, efficiently transporting genetic material and enhancing virulence and stability. The ASFV genome is 170–194 kilobase pairs and encodes over 150 open reading frames, allowing the expression of numerous proteins to adapt and evade host defenses. Based on the 3′-end sequences of the *B646L* gene, ASFV is classified into several genotypes [1], with genotype II being the most prevalent strain in China. First identified in Georgia in 2007 [2], genotype II ASFV caused widespread outbreaks in Eastern Europe and later spread to Asia. The emergence of genotype II in China [3] has been linked to extensive losses in the pig population, significantly impacting local farmers and the industry as a whole.

ASF is primarily transmitted through contact with infected animals, fomites, or uncooked pork products. It presents various clinical manifestations, including acute, subacute, chronic, and unapparent forms in domestic pigs [4]. There are currently no widely available, commercially licensed vaccines against ASF, so prevention relies on strict biosecurity practices. Vaccine research is ongoing, and various methods have been used to develop effective ASF vaccines, including inactivated vaccines, live attenuated vaccines (LAVs), DNA vaccines, subunit vaccines, and virus-vectored vaccines.

Early attempts to develop inactivated ASF vaccines have shown that although inactivated vaccines can induce some immunity, this response is often insufficient to prevent infection and viral replication. Despite efforts to enhance the efficacy of inactivated ASF vaccines by using a mild, BEI-inactivated ASFV preparation with a high antigen dose, simultaneous intradermal and intramuscular administration, a long-interval vaccination schedule, and strong modern adjuvants, the results did not provide effective protection against a lethal challenge [5]. Another study, designed to evaluate the efficacy of a gamma-irradiated inactivated ASF vaccine in combination with two state-of-the-art adjuvants, failed to provide protection and revealed a phenomenon of immune-mediated disease enhancement [6], further demonstrating the infeasibility of inactivated ASF vaccines.

Several research groups and institutions are actively developing LAVs against ASF. LAV use weakened forms of the virus, including natural attenuated, cell passage attenuated, and recombinant gene-deficient attenuated strains, that stimulate an immune response without causing the disease. Research on LAVs has mainly focused on gene-deficient strains, specifically those with deletions in genes related to viral genome replication and transcription (*K196R*, *QP509L*, *QP383R*, *A859L*, *E165R*, *C962R*, *A104R*, etc.), multigene family (MGF) genes (*MGF110-5L-6L*, *MGF360-12L*, *MGF360-13L*, *MGF360-14L*, *MGF505-1R*, *MGF505-2R*, *MGF505-3R*, etc.), hemadsorption-associated genes (*EP402R* and *EP153R*), and several genes with unknown function (*DP71L*, *DP96R*, *B119L*, *I177L*, *I226R*, *DP148R*, *A137R*, *H108R*, etc.) [7]. As LAVs, ASFV-G-ΔI177L and ASFV-G-ΔMGF have been approved in Vietnam. However, LAVs are not ideal vaccines due to the risks of reversion to virulence, genetic recombination, vertical transmission, and cross-protection [8].

Researchers are exploring DNA vaccines, subunit vaccines, and viral-vector vaccines to avoid the use of live viruses and enhance safety. Previous studies have shown that subunit vaccines can protect against genotype I lethal strains of ASFV [9,10,11]. However, an effective subunit vaccine against genotype II lethal strains has not yet been developed. Research on DNA vaccines has shown that they have limitations in their protective effects, likely due to the low levels of immune response they trigger. Therefore, DNA vaccines are often combined with other strategies, such as fusion expression with APCH1 or ubiquitin or by boosting with LVA to enhance the immune response [12,13,14,15]. Despite these combined strategies, protection against genotype I lethal strains of ASFV is still inadequate or offers only partial defense. When investigating protection against genotype II lethal strains, prime-boost strategies using DNA vaccines with recombinant poxvirus-vectored or recombinant subunit vaccines have not provided effective protection [16,17].

Several studies have shown that viral vectors delivering ASFV antigens can stimulate specific cellular immune responses and antibody-mediated humoral immune responses in pigs. This partially protects against lethal ASFV challenges [18,19,20,21]. Various viral vectors have been researched for use in ASFV vaccines. However, there is still a need for more studies to determine the most effective vector. While adenovirus (AdV), vaccinia virus, and baculovirus have been studied in swine, other viral vectors have only been tested in mouse models, leaving their effects on pigs still unknown. Newcastle Disease Virus (NDV) is a promising vaccine vector candidate for ASFV due to its safety and strong immunogenicity, with potential applications in both human and veterinary fields, including swine [22,23,24,25]. It has shown promise in developing vaccines against CSFV and PRRS in swine [26,27]. These results indicate that using an NDV-vectored vaccine against ASF is feasible; however, selecting the protective antigens for an effective immune response is challenging due to the genetic complexity and diversity of the virus. This study utilized NDV as a delivery platform to screen ASFV antigens. Four NDV-expressed antigens were selected to evaluate their safety, immunogenicity, and protective efficacy in swine.

## 2. Materials and Methods

### 2.1. Cells and Viruses

BSR T7/5 cells, the baby hamster kidney cell line BHK-21 that expresses T7 RNA polymerase, were cultured in Dulbecco’s Modified Eagle Medium (DMEM) supplemented with 10% fetal bovine serum (FBS), 100 U/mL penicillin, 100 μg/mL streptomycin, and 0.25 μg/mL amphotericin B in an incubator at 37 °C with 5% CO_2_. The pulmonary alveolar macrophages (PAMs) utilized in this study were prepared following a previously described methodology [28].

The NDV La Sota vaccine strain was maintained in our laboratory and propagated in specific-pathogen-free (SPF) embryonated chicken eggs that were 9 to 11 days old. The virulent genotype II ASFV-SY18 strain was isolated in our laboratory [3]. The genome of ASFV-SY18 (MH766894.2) was used as a reference sequence. The recombinant SY18∆I267L virus, in which an *EGFP* coding sequence replaced the *I267L* coding sequence, was obtained from our laboratory [29]. Both ASFV-SY18 and SY18∆I267L were cultured on pulmonary alveolar macrophages.

### 2.2. Plasmid Construction and Virus Rescue

A detailed description of the construction of the plasmid pcDNA3.1-rNDV, which contains the full-length cDNA of the NDV vaccine strain La Sota, has already been published [30,31,32,33]. Briefly, viral RNA was extracted using the Simple P Total RNA Extraction Kit (Catalog No. BSC52M1, Bioer, Shanghai, China) and transcribed into three overlapping complementary DNA (cDNA) fragments with the PrimeScript™ RT PCR Kit (Catalog No. RR014A, TaKaRa, Tokyo, Japan). The subgenomic cDNA fragments were cloned into a modified pcDNA3.1-HH plasmid using an infusion cloning kit (Catalog No. 638949, TaKaRa). The pcDNA3.1-HH plasmid included a CMV promoter, a self-cleaving hammerhead ribozyme (HamRz), and an autocatalytic hepatitis delta virus ribozyme (HDVRz), followed by a BGH polyA signal. A unique MluI site, along with the gene-start and gene-end sequence (GS-GE) of NDV La Sota, was introduced into the *P* and *M* intergenic regions to facilitate the insertion of the foreign ASFV gene. The open reading frame (ORF) of the ASFV genome, amplified via PCR from the genomic DNA of the ASFV-SY18 strain, was incorporated into the MluI site of the pcDNA3.1-rNDV plasmid. Subsequently, the recombinant pcDNA3.1-rNDV-ASFV plasmid and three supporting plasmids (pUC-NP, pUC-P, and pcDNA3.1-L), which encode the *NP*, *P*, and *L* antigenomes of NDV, were co-transfected into BSRT7/5 cells to generate recombinant NDV-vectored ASFV viruses (rNDV-ASFVs). After incubating for 10–16 h, the transfection mixture was replaced with a culture medium containing 1 μg/mL TPCK-treated trypsin (Catalog No. T1426, Sigma-Aldrich, St. Louis, MO, USA). Following the inoculation of the culture supernatant into the allantoic cavities of 9- to 11-day-old embryonated SPF eggs, an infectious rNDV-ASFV expressing the ASFV gene was rescued. A total of 131 pcDNA3.1-rNDV-ASFVs were constructed that expressed nearly all the ORFs of ASFV, but only 76 rNDV-ASFVs were successfully rescued. The rescued rNDV-ASFVs were detected by a hemagglutination (HA) assay with chicken erythrocytes. HA-positive allantoic fluid was validated by RT-PCR, confirming the integration of ASFV genes. Viral RNA was extracted from allantoic fluids and reverse-transcribed using EasyScript One-Step RT-PCR SuperMix (Catalog No. AT411, Transgene, Beijing, China), with a forward primer (5′-CCCACTGAATGATCGCGTAACCG-3′) and reverse primer (5′-CTAACAGGTTGCTAGAAGAATGGGCAG-3′). The resulting PCR products were analyzed on a 1% agarose gel. The 50% egg infective dose (EID50) of the virus was calculated according to the Reed and Muench method.

### 2.3. Western Blot Analysis Using Serum from Convalescent Pig Infected with ASFV-SY18

Serum from a convalescent pig infected with ASFV-SY18 was blotted against rNDV-ASFVs expressing ASFV proteins to characterize the serological immune dominants of ASFV. The detailed protocol followed routine experimental procedures for Western blot analysis. Briefly, BSRT7/5 cells in 6-well plates were infected with 76 rNDV-ASFVs encoding 76 ASFV ORFs, and uninfected BSRT7/5 cells served as a control. Cells exhibiting a cytopathic effect were lysed using a cell lysis buffer (Catalog No. P0013, Beyotime, Shanghai, China) 20–24 h post-infection and then centrifuged at 12,000 rpm for five minutes to collect the supernatant. The supernatant was mixed with 5× SDS loading buffer, heated for 10 min, and loaded onto 10–15% separating SDS-PAGE gels. After electrophoresis, the samples were transferred to polyvinylidene difluoride (PVDF) membranes (Catalog No. IPFL00010, Millipore, Billerica, MA, USA). To prevent non-specific binding of antibodies, the membrane was blocked overnight at 4 °C with 5% skim milk. It was then incubated with a 1:200 dilution of ASFV-SY18-infected convalescent pig serum in Primary Antibody Dilution Buffer (Catalog No. P0256, Beyotime) for 2 h at 37 °C, followed by three 5-min washes in TBST (25 mM Tris-HCl buffer at pH 8.0 containing 0.9% NaCl and 0.05% Tween-20). Subsequently, the membranes were incubated with horseradish peroxidase (HRP)-conjugated goat anti-swine antibodies at room temperature for one hour. Following a washing step to remove unbound antibodies, the membranes were detected with an electrochemiluminescence (ECL) substrate and analyzed using an imaging system.

### 2.4. Virus Neutralization

Eight-week-old SPF BALB/c female mice were randomly divided into 16 groups, each containing 8 mice. Each mouse received a 200 μL intramuscular injection. Fifteen groups were treated with different rNDV-ASFVs: rNDV-A104R, rNDV-A137R, rNDV-B602L, rNDV-B646L, rNDV-CP204L, rNDV-CP312R, rNDV-D117L, rNDV-E183L, rNDV-EP84R, rNDV-K145R, rNDV-K205R, rNDV-KP177R, rNDV-MGF110-5L-6L, and rNDV-O61R. The remaining group received the same volume of PBS as a control. The mice were inoculated every 14 days for a total of three doses and euthanized on day 42. Serum samples were collected for the ASFV neutralization assay, and spleen lymphocytes were obtained for the cell proliferation assay.

Mouse sera from rNDV-ASFV-immunized mice were evaluated for ASFV neutralization ability using a flow cytometry assay [34]. The experiment utilized ASFV-SY18ΔI267L, which expresses the fluorescent protein EGFP as a marker, facilitating the detection of ASFV-infected cells in vitro through fluorescent microscopy and flow cytometry assay. Heat-inactivated mouse sera were diluted fourfold with RPMI 1640 culture medium in 24-well plates, and an equal volume of 10^4^ TCID50/mL of SY18ΔI267L was added to each well. After incubating the serum–virus mixture for 2 h at 37 °C, PAMs were added. Cells were placed in a CO_2_ incubator for 4 days to allow for any virus replication. They were then examined under a fluorescence microscope. Subsequently, the cells were harvested and fixed overnight in 4% paraformaldehyde at 4 °C, followed by two washes with PBS. After resuspending in PBS with 2% FBS, the infected cells expressing EGFP were detected as FITC fluorescence by flow cytometry (CytoFLEX, Beckman, Miami, FL, USA). A scatter plot of FSC versus SSC was created to select the initial cell population. Next, the FITC channel was plotted, with EGFP fluorescence intensity on the X-axis and cell count on the Y-axis. The threshold for EGFP-positive cells was set based on the control group (non-EGFP-expressing), and a gate was defined around the EGFP-positive population. The percentage of cells within the gated area is calculated and displayed automatically by CytExpert (CytExpert2.2, Beckman, Brea, CA, USA). Virus neutralization (VN) was calculated with the following formula: VN = ((PI−) − (PI+))/PI−, where PI− is the percentage of infected cells treated with serum from mice immunized with PBS, and PI+ is the percentage of infected cells treated with serum from mice immunized with rNDV-ASFV [34].

### 2.5. Lymphocyte Proliferation Assay

Lymphocyte proliferation assays were employed to assess the capacity of the mouse spleen lymphocytes to proliferate in response to stimuli from the specific protein. Mouse spleen lymphocytes were isolated after euthanasia by mincing the spleen with a scalpel and filtering it through a cell strainer into a collection tube. After removal of the erythrocytes and rinsing with PBS, the cell pellets were resuspended and cultured in RPMI 1640 medium supplemented with 10% fetal bovine serum. For the proliferation assays, 5 × 10^5^ cells/mL of splenocytes were added to 96-well plates at a volume of 100 μL per well. The cells in each group were then stimulated with rAdV-ASFVs expressing the same specific ASFV proteins, including rAdV-A104R, rAdV-A137R, rAdV-B602L, rAdV-B646L, rAdV-CP204L, rAdV-CP312R, rAdV-D117L, rAdV-E183L, rAdV-EP84R, rAdV-K145R, rAdV-K205R, rAdV-KP177R, rAdV-MGF110-5L-6L, and rAdV-O61R. The PBS group was stimulated with rAdV-null. Concanavalin A (Con A; 5 μg/mL) and mock medium were added to groups to create positive and negative controls, respectively. Three days post-incubation, 10 μL of Cell Counting Kit-8 (CCK-8) (Catalog No. C0042, Beyotime) was added to each well and incubated for 4 h in a cell incubator. The optical density (OD) values were determined at 450 nm using a microplate reader (Bio-Rad, Hercules, CA, USA) with a reference value of 650 nm. The stimulation index (SI) was calculated using the following formula: SI = (OD450_value of the sample_ − OD650_value of the sample_)/(OD450_value of the negative control_ − OD650_value of the negative control_).

### 2.6. Swine Vaccination and Challenge

Landrace pigs weighing 15–20 kg were acquired from a local high-health farm. To determine the immunoprotective effect of the screened antigens, four pigs (172, 173, 174, and 175) were intramuscularly inoculated in the neck with a cocktail of rNDV-B602L, rNDV-EP84R, rNDV-KP177R, and rNDV-B646L, designated as the rNDV-ASFV group. Pigs 168, 169, and 170 served as the non-vaccinated control group. The rNDV-ASFV group of pigs received a total inoculation dose of 4 × 10^8^ EID50, comprising 10^8^ EID50 per gene. Two booster inoculations were administered to the pigs using the same vaccine formulation 14 days and 28 days post-prime. At 42 days post-prime, both the rNDV-ASFV and the control groups were challenged with ASFV-SY18 via intraoral (IO) infection at a dose of 2000 TCID50.

During the challenge phase, pigs were monitored daily for the clinical signs associated with ASF and scored as previously described [35]. Blood samples, oral swabs, and rectal swabs were collected on days 0, 7, and 14 post-challenge, as well as at the time of death. Various tissues, including the heart, liver, spleen, lungs, kidneys, intestines, thymus, tonsils, submandibular lymph nodes, and inguinal lymph nodes, were collected during necropsy. To monitor the immune response of the pigs post-inoculation and post-challenge, serum samples and porcine peripheral blood lymphocytes (PBMCs) were collected on days 0, 14, 28, 42, and 56.

### 2.7. qPCR for Viral Load Detection

The ASFV genome from the blood, swabs, and tissue samples of all pigs was extracted using the Virus DNA Extraction Kit (Catalog No. RC311, Vazyme, Nanjing, China) and detected by qPCR targeting the ASFV-encoded *B646L* gene (forward primer: 5′-GCTTTCAGGATAGAGATACAGCTCT-3′, reverse primer: 5′-CCGTAGTGGAAGGGTATGTAAGAG-3′, probe: FAM-CCGTAACTGCTCATGGTATCAATCTTATCG-BHQ1) on a LightCycler 480 II system (Roche, Basel, Switzerland) under the following conditions: 95 °C for 30 s, 40 cycles at 95 °C for 5 s, and 60 °C for 30 s, according to the manufacturer’s instructions (Catalog No. RR390, Takara). The quantification of the ASFV copy number was calculated following the standard curve established through the detection of the standard B646L plasmid.

### 2.8. Antibody Detection

A routine hemagglutination inhibition (HI) assay was used to measure NDV-specific antibodies. HI titers were reported as reciprocal log2 titers.

Antibodies against ASFV in pig serum were detected using a commercial blocking enzyme-linked immunosorbent assay (ELISA) kit targeting the p72 protein (Catalog No. ASF.K001/5, Ahreal, Qingdao, China). In summary, 50 μL of diluent and 50 μL of serum samples, including positive and negative controls, were added to wells coated with p72 protein. After overnight incubation at 20–25 °C, the wells were washed, and 100 μL of conjugate solution was added for an additional 30 min. The wells were washed three more times, and substrate solution was added, followed by the stop solution. The OD value was measured at 450 nm. The results were interpreted based on the percentage of inhibition (PI), calculated using the formula X = (OD_negative control_ − OD_sample_)/(OD_negative control_ − OD_positive control_).

### 2.9. IFN-γ Enzyme-Linked ImmunoSpot Assay

The quantity of porcine IFN-γ-secreting T-cells was determined by an IFN-γ enzyme-linked ImmunoSpot (ELISPOT) assay with a commercial kit (Catalog No. pIFNg-1M, ImmunoSpot, Cleveland, OH, USA) to evaluate the cellular immune response to ASFV infection. Each well received 5 × 10^5^ PBMCs and 10^5^ TCID50 ASFV-SY18 stimulants to induce IFN-γ secretion. A mock medium served as the negative control, while ConA (4 μg/mL) acted as the positive control. After a 24-h incubation, anti-porcine IFN-γ detection antibodies were added, followed by a substrate that reacts to form spots. A CTL ImmunoSpot reader was used to visualize and count these spots. The results were expressed as the number of antigen-specific IFN-γ spot-forming cells per 10^6^ PBMCs, with background medium counts subtracted.

### 2.10. Cytokine Detection

Cytokine levels were assessed using sandwich ELISA, measuring tumor necrosis factor-alpha (TNF-α), interleukin-1 beta (IL-1β), interleukin-12 (IL-12), and interleukin-10 (IL-10) with commercial kits (Catalog Nos. PTA00, PLB00B, P1240, and P1000, R&D Systems, Minneapolis, MN, USA). All assays were performed in strict accordance with the manufacturer’s instructions, and cytokine concentrations were derived from independently constructed standard curves for each assay.

### 2.11. Statistical Analysis

Data were expressed as the mean with standard deviation (SD). All statistical analyses were performed using the parametric unpaired *t*-test via GraphPad Prism (GraphPad Prism 8, La Jolla, CA, USA). A significance threshold was set at a *p*-value of ≤0.05, marked with an asterisk (*). If the *p*-value falls between 0.05 and 0.10, it indicates a statistical tendency, denoted by two asterisks (**). Highly significant results, where the *p*-value is between 0.05 and 0.001, are indicated with three asterisks (***).

### 2.12. Biosafety Management and Ethics Statement

The swine challenge experiments were implemented in an animal biosafety level 3 laboratory at the Changchun Veterinary Research Institute (CVRI), Chinese Academy of Agricultural Sciences, approved by the Ministry of Agriculture and Rural Affairs. All animal experiments were performed according to standard operating procedures approved by the Laboratory Animal Welfare Committee of CVRI (Review ID: IACUC of CAS-12-2021-011, approved on 1 December 2021) and conducted in strict accordance with the recommendations in the Guide for the Care and Use of Laboratory Animals of the Ministry of Science and Technology of the People’s Republic of China. Every effort was made to minimize animal pain, suffering, and distress while reducing the number of animals used.

## 3. Results

### 3.1. Construction and Rescue of rNDV-ASFVs Expressing ASFV Antigens

**Figure 1:**
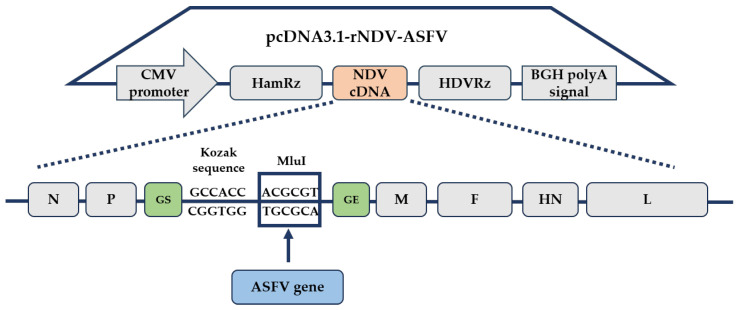
Structure of the plasmid pcDNA3.1-rNDV-ASFV. The full-length cDNA of the Newcastle Disease Virus (NDV) vaccine strain La Sota was assembled in pcDNA3.1-HH, a modified plasmid featuring a CMV promoter, a hammerhead ribozyme (HamRz), a hepatitis delta virus ribozyme (HDVRz), and a BGH polyA signal. A gene-start and gene-end sequence (GS-GE), comprising the Kozak sequence and MluI site, was incorporated into the intergenic region between the *P* and *M* genes of NDV cDNA. The open reading frame (ORF) of the African Swine Fever Virus (ASFV) genome was introduced at the MluI site to construct the plasmid pcDNA3.1-rNDV-ASFV.

**Figure 2:**
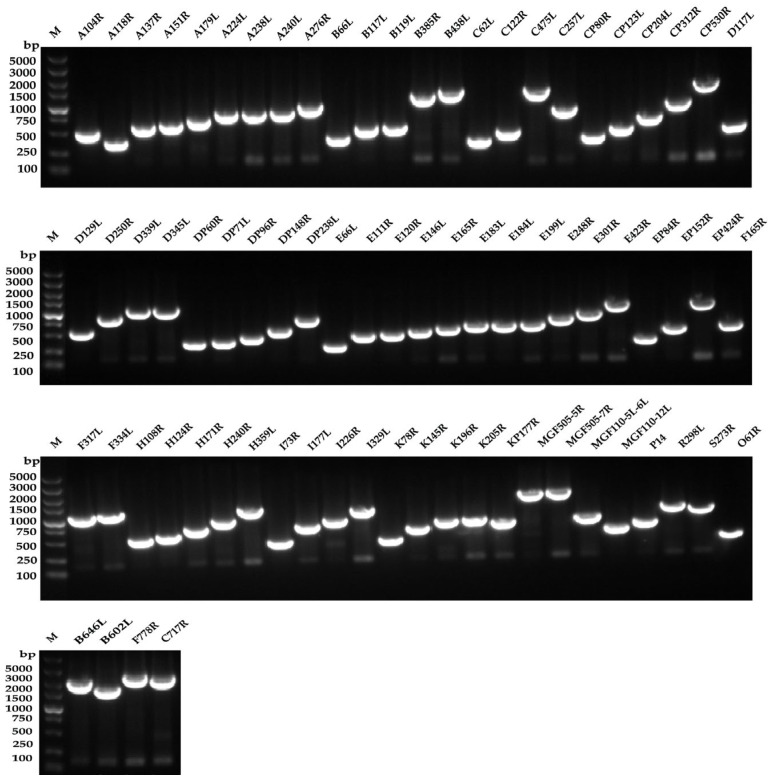
The presence of ASFV genes in rNDV-ASFV was confirmed using RT-PCR with primers targeting the ASFV gene. M indicates the DNA marker, followed by the names of the ASFV genes.

The ASFV gene was inserted into the intergenic region between the P and M genes of NDV La Sota’s cDNA to construct pcDNA3.1-rNDV-ASFV (Figure 1). The full-length cDNA of rNDV-ASFV follows the “rule of six”. The rNDV-ASFVs were rescued by co-transfecting pcDNA3.1-rNDV-ASFV and three supporting plasmids (pUC-N, pUC-P, and pcDNA3.1-L) into BSR-T7/5 cells and then propagated in 9- to 11-day-old embryonated SPF eggs. The rescue of the rNDV-ASFVs was verified by an HA assay. To confirm the presence of the ASFV gene in the rNDV-ASFV genome, viral RNA was extracted from allantoic fluids and analyzed by RT-PCR using primers flanking the ASFV gene. All 76 rNDV-ASFVs produced PCR products corresponding to the target ASFV gene segments (Figure 2), confirming successful transgene insertion between the *P* and *M* genes of rNDV. The sizes of the ASFV genes are shown in Table S1 (see Supplementary Materials).

### 3.2. Identification of ASFV-Immunodominant Antigens by Western Blot Analysis

**Figure 3:**
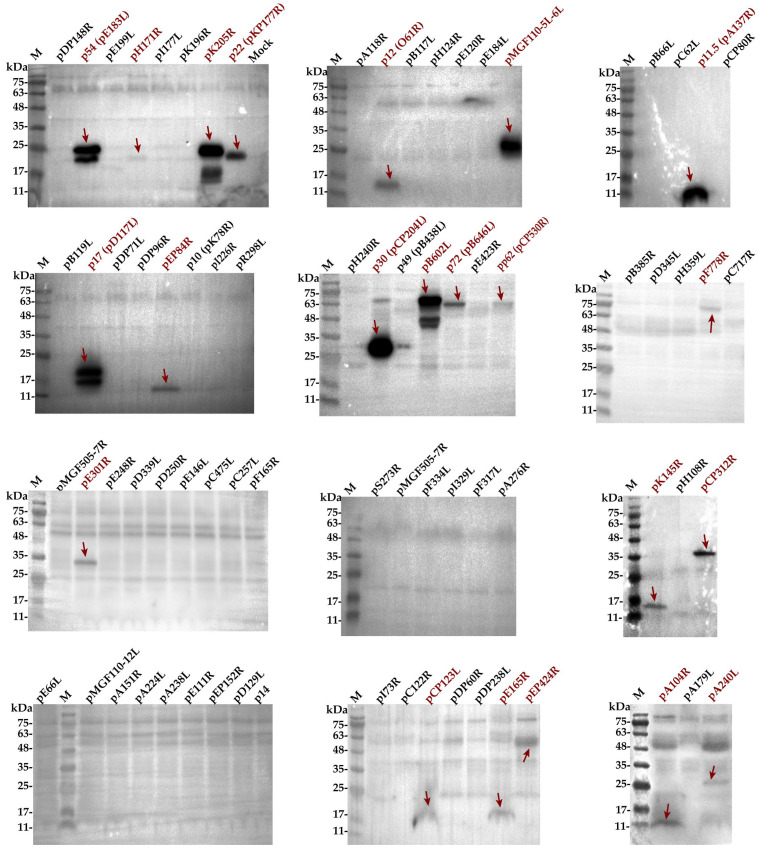
Reaction of rNDV-ASFV-expressed antigens with serum from a convalescent pig infected with ASFV-SY18. M indicates the protein marker, followed by the names of the ASFV proteins expressed by rNdV-ASFVs and their corresponding gene names in parentheses. Positive bands are marked with dark-red arrows.

To identify the immunodominant antigens in ASFV infection, we analyzed ASFV antigens expressed by rNDV-ASFVs using Western blot analysis with serum from a convalescent pig infected with ASFV-SY18 strain. A total of 76 proteins expressed by rNDV-ASFVs reacted to varying degrees with the serum, as shown in Figure 3. Notably, p30 (pCP204L) elicited the strongest antibody response during ASFV infection. Other proteins such as p54 (pE183L), pK205R, pMGF110-5L-6L, p11.5 (pA137R), p17 (pD117L), and pB602L also reacted positively. p22 (pKP177R), p12 (pO61R), pEP84R, p72 (pB646L), pK145R, pCP312R, and pA104R showed a weak positive reaction. Additionally, pH171R, pp62 (pCP530R), pF778R, pE301R, pCP123L, pE165R, pEP424R, and pH240L exhibit faint but detectable bands consistent with their theoretical molecular weights. The remaining 54 proteins did not react with the serum. The sizes of the ASFV proteins are shown in Table S1 (see Supplementary Materials). The results show that 14 ASFV proteins, including p30 (pCP204L), p54 (pE183L), pK205R, pMGF110-5L-6L, p11.5 (pA137R), p17 (pD117L), pB602L, p22 (pKP177R), p12 (pO61R), pEP84R, p72 (pB646L), pK145R, pCP312R, and pA104R, displayed antigenicity, indicating they are likely the main ASFV antigens capable of provoking effective immune responses.

### 3.3. Screening of Key ASFV Antigens Involved in Immune Detection

**Figure 4:**
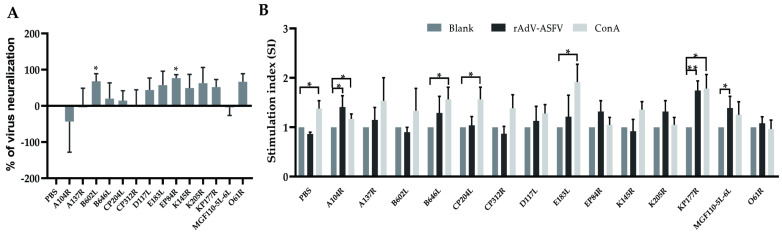
Immune response in mice after injection with rNDV-ASFVs expressing immunodominant antigens. (**A**) Virus neutralization (VN) is expressed as the percentage of positive serum relative to negative PBS serum, with the latter indicating a neutralization value of 0. Values above the baseline indicate a neutralizing effect against the virus, while values below the baseline may suggest enhanced viral proliferation. (**B**) ASFV antigen-specific T-cell proliferation of mice splenic lymphocytes was tested by a Cell Counting Kit-8 assay. Cells were stimulated with rAdV-ASFVs expressing the same ASFV proteins as rNDV-ASFVs, while ConA stimulation served as a control. The data are presented as the mean with SD. Comparisons were made using the parametric unpaired *t*-test. * *p* < 0.05; ** *p* < 0.01.

To further investigate the potential immunoprotective ASFV antigens, we inoculated mice with rNdV-CP204L, rNdV-E183L, rNdV-K205R, rNdV-MGF110-5L-6L, rNdV-A137R, rNdV-D117R, rNdV-B602L, rNdV-KP177R, rNdV-O61R, rNdV-EP84R, rNdV-B646L, rNdV-K145R, rNdV-CP312R, and rNdV-A104R, expressing ASFV-immunodominant proteins. Serum samples were then tested for neutralizing effects using a neutralization assay. All sera from the inoculated groups, except those from rNdV-A104R and rNdV-MGF110-5L-6L, demonstrated partial neutralization of SY18ΔI267L, although none achieved complete neutralization (Figure 4A). Notably, significant neutralizing activity was seen in the sera from the rNdV-EP84R and rNdV-B602L groups (*p* < 0.05) (Figure 4A). This suggests that the antibodies elicited by the pEP84R and pB602L may be critical in developing effective immunoprotective strategies against ASFV.

Antigen-specific T-cell proliferative responses were detected to study the cellular immune responses induced by rNDV-ASFVs. After euthanasia, splenic cells from the mice were collected and stimulated with ConA or rAdV-ASFVs expressing the same ASFV proteins. Stimulation with ConA resulted in significant cell proliferation in the rNdV-A104R, rNdV-B646L, rNdV-CP204L, rNdV-E183L, and rNdV-KP177R groups (*p* < 0.05) (Figure 4B). When stimulated with rAdV-ASFV expressing the corresponding specific ASVF antigens, significant proliferation was observed in the rNdV-A104R and the rNdV-MGF110-5L-6L groups (*p* < 0.05). In addition, the rNdV-KP177R group showed an even stronger response following stimulation with rAdV-KP177R (*p* < 0.01). Other groups showed a slight tendency to increase after stimulation with either ConA or rAdV-ASFVs but did not differ significantly from the blank control. The notable response in the rNdV-KP177R group suggests that pKP177R may induce a cellular immune response in vivo.

### 3.4. Inoculation of Pigs with rNDV-ASFV Cocktail Delays Disease Progression

**Figure 5:**
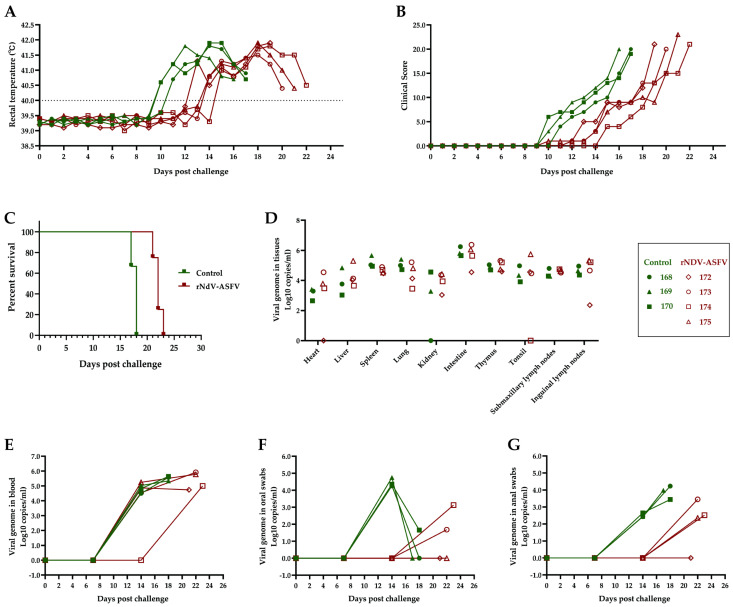
Clinical and virological parameters in the pigs post-challenge. Rectal temperature (**A**) and the clinical score (**B**) were measured for each pig. The dotted line represents the normal rectal temperature for pigs (40.0 °C); a temperature exceeding 40.0 °C is classified as fever. (**C**) Survival rates were evaluated by a Kaplan–Meier curve. Different tissues from autopsies (**D**) as well as blood (**E**), oral swabs (**F**), and anal swabs (**G**) collected at different time points were tested for ASFV viral load. The green lines in (**A**,**B**,**E**–**G**) represent the control group, while the red lines represent the rNDV-ASFV group.

The capsid protein p72, encoded by the B646L gene, is the major structural component of the ASFV, accounting for 31–33% of its mass and serving as the main antigenic protein. In our experiment, a cocktail of rNDV-B646L and other potential ASFV immunoprotective antigens (rNDV-B604L, rNDV-EP84R, and rNDV-KP177R) was utilized to assess their protective effects in pigs. The pigs received inoculations every 14 days, for a total of three doses, and were then infected with ASFV-SY18 through oral immunization. Eight days post-challenge, the control pigs exhibited notable clinical symptoms, including fever, loss of appetite, mental depression, and cutaneous erythema, with all dying on day 16 or 17 (Figure 5A–C). In contrast, the rNDV-ASFV group showed delayed symptom onset, with changes appearing on day 12, and all pigs died on days 19 or 21, indicating a 3–4 day delay in disease progression (Figure 5A–C). By day 14, three pigs in the control group showed signs of viremia, while all but one in the rNDV-ASFV group remained undetectable (pig 174) (Figure 5E). Further analysis of oral and anal swabs on day 14 from the control group revealed detectable levels of ASFV in both body secretions. Conversely, the rNDV-ASFV group had no detectable virus in either oral or anal samples (Figure 5F,G). Tissue analysis (heart, liver, spleen, lungs, kidneys, intestines, thymus, tonsils, submandibular, and inguinal lymph nodes) showed no significant differences in viral load between the rNDV-ASFV and control groups at death (Figure 5D). While the rNDV group did not provide complete protection, the results indicate its effectiveness in delaying disease progression.

### 3.5. Antibody and Cellular Immune Responses in Pigs Inoculated with rNDV-ASFV Cocktail

**Figure 6:**
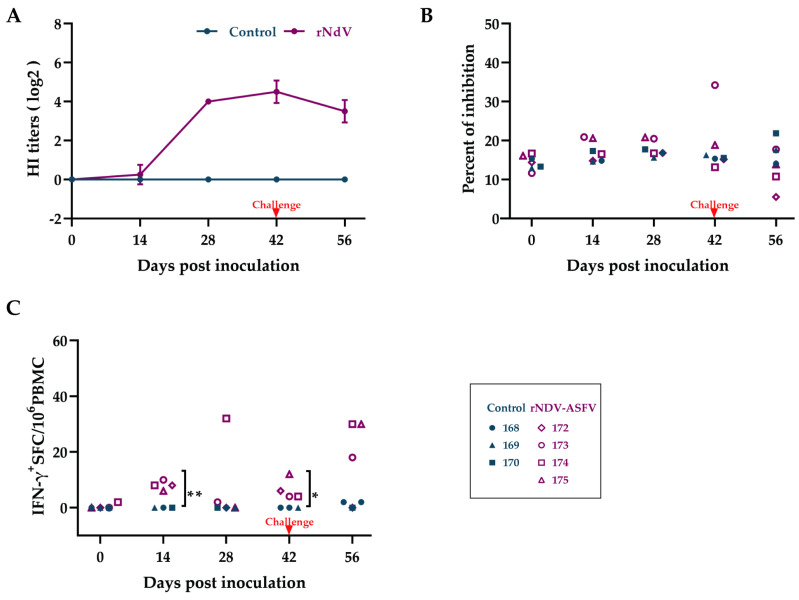
Immune responses post-inoculation and post-challenge in pigs. (**A**) Antibodies against NDV in porcine serum samples were detected by an HI assay. (**B**) Antibodies to the ASFV p72 were assessed by a blocking ELISA, with samples showing a percent of inhibition (PI) of < 40% classified as negative. (**C**) ASFV-specific IFN-γ-secreting PBMC responses were detected by ELISPOT. The data are presented as the mean with SD. Comparisons were made using the parametric unpaired *t*-test. * *p* < 0.05; ** *p* < 0.01.

Evaluating the humoral and cellular immune response against ASFV by measuring NDV antibodies, ASFV-specific p72 antibodies, and ASFV-specific IFN-γ-secreting cells provided a comprehensive understanding of the host’s defense mechanisms. The HI assay was employed to assess the antibody response to NDV (Figure 6A). NDV antibodies were undetected at day 14 post-prime but significantly increased by day 28 after the first boost, remaining stable at days 42 and 56. These results demonstrate the efficacy of the NDV vector in inducing a strong immune response. However, there was no change in the detection of specific antibodies against ASFV p72 (Figure 6B).

Cellular immunity against ASFV was assessed by the activity of ASFV-specific IFN-γ-secreting cells (Figure 6C). The rNDV-ASFV group had significantly higher levels of IFN-γ-secreting PBMCs as early as 14 days post-prime. By day 28, pig 174 showed a significant increase in IFN-γ-secreting PBMCs after the first boost, while others showed minimal responses. By day 42, 14 days after the second boost, the rNDV-ASFV group exhibited a higher level of IFN-γ-secreting PBMCs compared to the control group. Following the challenge, all rNDV-ASFV pigs, except pig 172, had significantly elevated IFN-γ-secreting PBMC levels. These results indicate that the rNDV-ASFV group successfully induced a cellular immune response, albeit at a low level.

### 3.6. Cytokine Changes in Pigs Inoculated with rNDV-ASFV Cocktail

**Figure 7:**
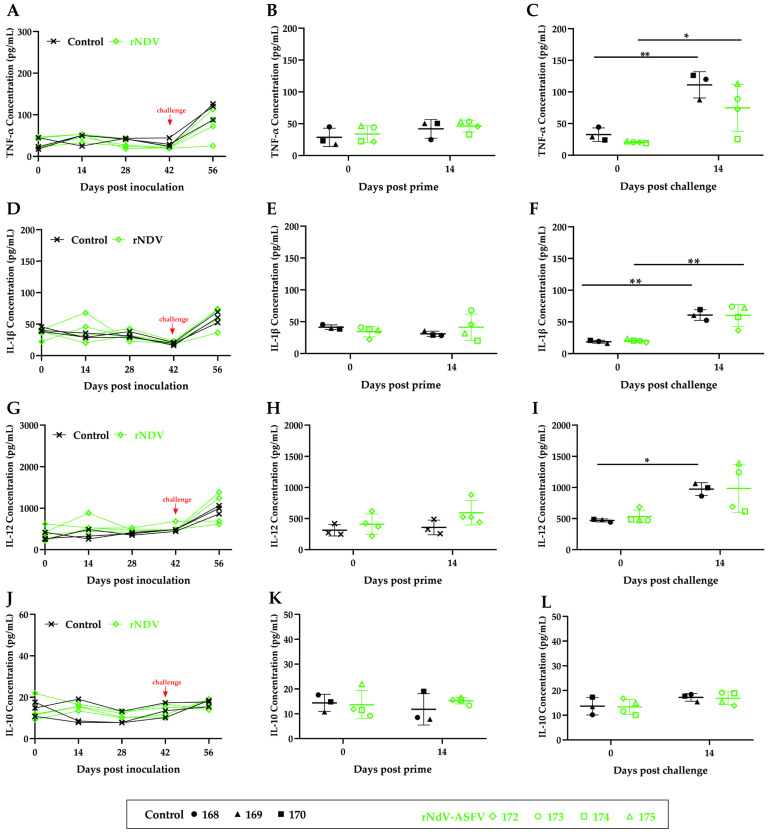
The kinetics of serum cytokines. The concentrations of the pro-inflammatory cytokines TNF-α (**A**–**C**), IL-1β (**D**–**F**), and IL-12 (**G**–**I**) as well as the anti-inflammatory cytokine IL-10 (**J**–L) were quantified using ELISA. Panels (**B**,**E**,**H**,**K)** show the levels of TNF-α, IL-1β, IL-12, and IL-10 before inoculation and 14 days post-prime. Panels (**C**,**F**,**I**,**L**) show the levels before the challenge and 14 days post-challenge. The data are presented as the mean with SD. Comparisons were made using the parametric unpaired *t*-test. * *p* < 0.05; ** *p* < 0.01.

To study cytokine changes following inoculation and challenge, levels of pro-inflammatory cytokines TNF-α, IL-1β, IL-12, and the anti-inflammatory cytokine IL-10 were measured (Figure 7). TNF-α remained stable post-inoculation but increased significantly post-challenge in both the rNDV-ASFV and control groups, with a more pronounced rise in the control group. IL-1β also remained stable post-inoculation in both groups but increased significantly post-challenge. IL-12 showed no significant changes post-inoculation in either group but increased significantly in the control group and partially in the rNDV-ASFV group post-challenge. In contrast, the anti-inflammatory cytokine IL-10 showed no changes post-inoculation and post-challenge. The results suggest that an increase in pro-inflammatory cytokines without a corresponding change in anti-inflammatory cytokines may create a cytokine imbalance, potentially leading to excessive inflammation that exacerbates the disease.

## 4. Discussion

In developing virus-vectored vaccines, the selection of antigens and the generation of effective immune responses are crucial for success. Early studies targeted antigens like p30, p54, and p72, but their findings indicate that the antibody responses they elicit are inadequate for full protection [36]. To improve vaccine efficacy, researchers have explored a variety of strategies for antigen screening, focusing on identifying antigens with the highest immunoprotective potential. Three primary methodologies have emerged: (1) screening based on gene functions, through which Lokhandwala assessed the immunogenicity of seven ASFV vaccine candidates [37], upon which Jancovich expanded by cloning 47 ASFV genes into plasmids and recombinant vaccinia viruses for immunization [16]; (2) immunoinformatics for identifying immune epitopes, with Lopera-Madrid using the Vaxign tool to analyze twelve ASFV genomes for potential immunogenic proteins [38] and Cadenas-Fernández employing the NetMHCpan4.0 algorithm to predict nine genes likely to bind strongly to swine leukocyte antigen (SLA) class I alleles, thus targeting those that can provoke a robust immune response [33]; and (3) in vitro screening for immune response activation, exemplified by Netherton’s use of an IFN-γ ELISPOT assay to identify eighteen antigens recognized by lymphocytes in ASF-immune pigs from a pool of 133 viral proteins [39]. Rodríguez’s group conducted immunopeptidomic studies that identified fifteen ASFV antigens recognized by ASFV-specific CD8+ T-cells, based on SLA class I peptide data [15,40]. Additionally, Zajac validated a broad array of antigens using convalescent serum from ASF-infected pigs via flow cytometry [20]. Although these studies have identified that the genes screened above can partially stimulate pigs’ humoral and cellular immune responses, they have not resulted in adequate protection, indicating a need for further experimental verification to find antigens that can elicit a robust immune protective response.

In this study, we utilized NDV as a vector to generate 76 rNDVs expressing ASFV proteins without tags to preserve their structural integrity. Screening with sera from a pig recovered from natural ASFV-SY18 infection identified 22 proteins positive in Western blot tests, while the other 54 did not react, potentially due to various factors. Some proteins might have low immunogenicity, resulting in weak antibody responses after ASFV infection. For example, Xu’s study indicated that 11 of 35 ASFV structural proteins expressed in E. coli were unrecognized by sera from ASFV-infected pigs, consistent with our results [41]. Various factors also influence ASFV antibody production and can affect immunological outcomes. Immune responses can vary by ASFV strain; for instance, sera from pigs infected with Malawi and Malta strains recognized pK196R, while those from ASFV-SY18 strain infections did not [41,42]. Significant differences in antibody production have been noted among pig breeds, as well as between outbred and inbred pigs, and between sows and finishing pigs [39,43]. Additionally, our use of a denaturing Western blot method focused on linear epitopes, while some proteins are recognized through conformational epitopes. For example, Borca found that amino acid residues 400 to 404 of p72 exhibited conformational dependence in reaction with its monoclonal antibody mAb 135D4 [44]. Similarly, while ELISA can detect pF334L, it did not yield positive results in Western blot analysis, likely due to the necessity for conformation-dependent epitopes [42]. Lastly, variations in protein expression levels can occur, even when RNA levels appear similar, leading to differences in antibody recognition.

We selected rNDV-EP84R, rNDV-B602L, and rNDV-KP177R, identified through neutralization experiments and mouse spleen lymphocyte proliferation tests, for immunization experiments in pigs. To improve immune response, we included rNDV-B646L, which encodes the capsid protein p72, a significant component of ASFV, making up one-third of its total protein. However, we found no antibodies against p72 in pigs following priming and two boosts, aligning with Goatley’s findings that pigs immunized with a pool of eight virus-vectored ASFV genes (*B602L*, *B646L*, *CP204L*, *E183L*, *E199L*, *EP153R*, *F317L*, and *MGF505-5R*) also failed to produce anti-p72 antibodies after immunization and challenge. Only two surviving pigs exhibited anti-p72 antibodies 20 days post-challenge, while antibodies against pB602L, p30 (pCP204L), and p54 (pE183L) were detected post-immunization. Even with higher doses of immunization, antibodies against p72 were not generated [19]. The absence or low levels of antibody response towards p72 have also been supported by other studies [18,38,45]. Interestingly, Liu’s research demonstrated that Ad2-p72-p72c, which expresses p72 and its chaperone protein pB602L, effectively induced anti-p72 antibodies [46]. This suggests that pB602L facilitates the correct folding of p72, enabling the immune system to recognize its conformational epitopes properly. Studies on inactivated ASF vaccines show that maintaining the structural integrity of viral antigens can induce anti-p72 antibodies, reinforcing the idea that anti-p72 recognition is primarily conformational-epitope-specific [6,47]. Hence, when developing vector, subunit, or DNA vaccines for p72, the co-expression of the chaperone protein pB602L may be essential for generating specific anti-p72 antibodies.

Understanding the porcine immune response to ASFV is crucial for vaccine development, particularly regarding the contentious role of anti-ASFV antibodies. Studies indicate that the passive transfer of serum or colostrum from convalescent pigs can delay symptoms, reduce viremia, and enhance survival in ASFV-infected pigs, suggesting the importance of humoral immunity in combating ASFV infection [48,49]. Studies on LVA vaccination indicate that anti-ASFV antibodies are associated with protection against lethal ASFV challenges [50,51]. Conversely, other vaccines—such as inactivated, DNA, subunit, and vector vaccines—while generating anti-ASFV antibodies, have failed to provide sufficient protection against lethal ASFV [10,15,17,20,36,47]. A notable study by Luong revealed that pigs vaccinated with LAVs survived virulent ASFV infection, whereas eight of ten pigs in the inactivated virus group died within 14 days post-challenge, despite similar levels of anti-p72 antibodies, which may be related to the fact that the LAVs triggered antibody responses against a broader range of viral proteins compared to the inactivated vaccine [52]. Collectively, these findings suggest that sera from convalescent pigs with LAVs or virulent ASFV infection yield stronger antibody responses targeting a wider array of viral proteins, whereas vector vaccines may offer weaker protection due to a limited selection of antigens. Additionally, the concern of antibody-dependent enhancement (ADE) in vector vaccine studies persists, although understanding of this phenomenon remains lacking [17,18,39].

Relying solely on ASFV-specific antibodies is insufficient for effective protection against virulent ASFV. Cellular immune responses play an indispensable role in the protective immunity against ASFV [53,54,55]. Our experiment observed that the rNDV-ASFV group exhibited a significant ASFV-specific IFN-γ-secreting cell response compared to the control group, especially in pigs 174 and 175, which had a stronger cellular response and later onset of clinical symptoms. King’s study supported this, showing that the ability of different ASFV isolates to stimulate IFN-γ production from the immune pig lymphocytes correlates with the ability to induce cross-protection against different isolates [56]. Oura demonstrated that depleting CD8+ lymphocytes diminished immune defense against virulent ASFV [53]. The findings indicate that the ASFV-specific IFN-γ-producing T-cell response is linked to protective efficacy against virulent ASFV infection. Consequently, the low levels of IFN-γ-producing T-cells in the rNDV-ASFV group’s splenic lymphocytes may have impaired immune protection.

Furthermore, ASFV has a complex antigenic structure, with multiple proteins that can elicit different immune responses. Some antigens can induce strong cellular immunity but weak antibody responses [39], while others may lead to strong antibody responses with weak cellular immune reactions [15,38]. This variation may be linked to the different B- and T-cell epitopes and the immune system can produce distinct responses to different antigen presentations [57]. Additionally, research into ASFV proteins and their interactions with the antiviral immune response reveals that ASFV suppresses immune response by regulating interferon signaling, manipulating inflammatory pathways, and influencing apoptosis [58,59]. Therefore, possessing a deep understanding of the relationship among ASFV antigens, neutralizing antibodies, and cellular immune responses, as well as exploring gene combinations that jointly induce neutralizing antibodies and cellular immune responses, is crucial for designing an ASF vaccine with protective efficacy.

In addition to investigating the effects of humoral and cellular immunity on protection against ASFV, we also examined changes in cytokines. The rNDV-ASFV group exhibited minimal cytokine level alterations post-inoculation, demonstrating the vector’s safety and the absence of inflammatory response induction. However, shortly after the challenge, there was a significant increase in pro-inflammatory cytokines TNF-α, IL-1β, and IL-12, while the anti-inflammatory cytokine TNF-10 remained unchanged. This led to an overexpression of inflammatory factors and a cytokine imbalance, consistent with Wang’s findings of a cytokine storm following lethal ASFV infection [60]. Such an imbalance can trigger acute inflammation, a critical factor in ASFV pathogenesis, as Zuo has explained [61]. While inflammatory response inhibition can somewhat improve clinical symptoms, it does not seem to change the lethal outcome of ASFV infection [19,21]. Efforts to reduce inflammation and prevent disease progression include Jiao’s study, which found that the IFN cocktail modulated pro- and anti-inflammatory cytokine production, mitigating tissue injury and delaying disease onset in ASFV-infected pigs [62]. Similarly, Jackman’s research identified tetrandrine and berbamine as strong antiviral agents against ASFV from a screening of 297 natural anti-inflammatory compounds, significantly lowering pro-inflammatory cytokines in infected cells [63]. In conclusion, using adjuvants or immune-modulatory materials like the IFN cocktail and selected anti-inflammatory compounds may help control cytokine production, potentially preventing disease progression and reducing ASF-related mortality. This study indicates that while vector vaccines for ASF show promise, challenges such as antigen selection, sustaining humoral and cellular immune responses, and managing cytokine storms require further research.

## 5. Conclusions

This study is the first to utilize an rNDV-vectored cocktail of ASFV antigens for immunizing pigs, providing a thorough evaluation of humoral, cellular, and cytokine responses to gain a comprehensive understanding of immune effects. It confirms the safety of the rNDV vector in pigs, with no cytokine imbalances observed post-inoculation. While the vaccine successfully delayed disease onset, it did not confer protection, and the immune responses were relatively weak. Future research should aim to identify protective ASFV antigens and enhance immune responses in pigs to improve vaccine efficacy.

## Data Availability

The original contributions presented in this study are included in the article/Supplementary Material. Further inquiries can be directed to the corresponding authors.

## Supplementary Materials

The following Supporting Information can be downloaded at https://www.mdpi.com/article/10.3390/microorganisms12122590/s1, Table S1: Sizes of the constructed ASFV genes and expressed proteins.

## References

[1] Bastos A.D.S., Hutchings G., Roger F., Couacy-Hymann E., Thomson G.R. (2003). Genotyping Field Strains of African Swine Fever Virus by Partial p72 Gene Characterisation. Arch. Virol..

[2] Rowlands R.J., Michaud V., Heath L., Hutchings G., Oura C., Vosloo W., Dwarka R., Onashvili T., Albina E., Dixon L.K. (2008). African Swine Fever Virus Isolate, Georgia, 2007. Emerg. Infect. Dis..

[3] Zhou X., Li N., Luo Y., Liu Y., Miao F., Chen T., Zhang S., Cao P., Li X., Tian K. (2018). Emergence of African Swine Fever in China, 2018. Transbound. Emerg. Dis..

[4] Blome S., Gabriel C., Beer M. (2013). Pathogenesis of African Swine Fever in Domestic Pigs and European Wild Boar. Virus Res..

[5] Cadenas-Fernández E., Sánchez-Vizcaíno J.M., van den Born E., Kosowska A., van Kilsdonk E., Fernández-Pacheco P., Gallardo C., Arias M., Barasona J.A. (2021). High Doses of Inactivated African Swine Fever Virus Are Safe, but Do Not Confer Protection against a Virulent Challenge. Vaccines.

[6] Pikalo J., Porfiri L., Akimkin V., Roszyk H., Pannhorst K., Kangethe R.T., Wijewardana V., Sehl-Ewert J., Beer M., Cattoli G. (2022). Vaccination with a Gamma Irradiation-Inactivated African Swine Fever Virus Is Safe But Does Not Protect Against a Challenge. Front. Immunol..

[7] Vu H.L.X., McVey D.S. (2024). Recent Progress on Gene-Deleted Live-Attenuated African Swine Fever Virus Vaccines. NPJ Vaccines.

[8] Urbano A.C., Ferreira F. (2022). African Swine Fever Control and Prevention: An Update on Vaccine Development. Emerg. Microbes Infect..

[9] Ruiz-Gonzalvo F., Rodríguez F., Escribano J.M. (1996). Functional and Immunological Properties of the Baculovirus-Expressed Hemagglutinin of African Swine Fever Virus. Virology.

[10] Gómez-Puertas P., Rodríguez F., Oviedo J.M., Brun A., Alonso C., Escribano J.M. (1998). The African Swine Fever Virus Proteins P54 and P30 Are Involved in Two Distinct Steps of Virus Attachment and Both Contribute to the Antibody-Mediated Protective Immune Response. Virology.

[11] Barderas M.G., Rodríguez F., Gómez-Puertas P., Avilés M., Beitia F., Alonso C., Escribano J.M. (2001). Antigenic and Immunogenic Properties of a Chimera of Two Immunodominant African Swine Fever Virus Proteins. Arch. Virol..

[12] Argilaguet J.M., Pérez-Martín E., Gallardo C., Salguero F.J., Borrego B., Lacasta A., Accensi F., Díaz I., Nofrarías M., Pujols J. (2011). Enhancing DNA Immunization by Targeting ASFV Antigens to SLA-II Bearing Cells. Vaccine.

[13] Argilaguet J.M., Pérez-Martín E., Nofrarías M., Gallardo C., Accensi F., Lacasta A., Mora M., Ballester M., Galindo-Cardiel I., López-Soria S. (2012). DNA Vaccination Partially Protects against African Swine Fever Virus Lethal Challenge in the Absence of Antibodies. PLoS ONE.

[14] Lacasta A., Ballester M., Monteagudo P.L., Rodríguez J.M., Salas M.L., Accensi F., Pina-Pedrero S., Bensaid A., Argilaguet J., López-Soria S. (2014). Expression Library Immunization Can Confer Protection against Lethal Challenge with African Swine Fever Virus. J. Virol..

[15] Bosch-Camós L., López E., Collado J., Navas M.J., Blanco-Fuertes M., Pina-Pedrero S., Accensi F., Salas M.L., Mundt E., Nikolin V. (2021). M448R and MGF505-7R: Two African Swine Fever Virus Antigens Commonly Recognized by ASFV-Specific T-Cells and with Protective Potential. Vaccines.

[16] Jancovich J.K., Chapman D., Hansen D.T., Robida M.D., Loskutov A., Craciunescu F., Borovkov A., Kibler K., Goatley L., King K. (2018). Immunization of Pigs by DNA Prime and Recombinant Vaccinia Virus Boost to Identify and Rank African Swine Fever Virus Immunogenic and Protective Proteins. J. Virol..

[17] Sunwoo S.-Y., Pérez-Núñez D., Morozov I., Sánchez E., Gaudreault N., Trujillo J., Mur L., Nogal M., Madden D., Urbaniak K. (2019). DNA-Protein Vaccination Strategy Does Not Protect from Challenge with African Swine Fever Virus Armenia 2007 Strain. Vaccines.

[18] Lokhandwala S., Petrovan V., Popescu L., Sangewar N., Elijah C., Stoian A., Olcha M., Ennen L., Bray J., Bishop R.P. (2019). Adenovirus-Vectored African Swine Fever Virus Antigen Cocktails Are Immunogenic but Not Protective against Intranasal Challenge with Georgia 2007/1 Isolate. Vet. Microbiol..

[19] Goatley L.C., Reis A.L., Portugal R., Goldswain H., Shimmon G.L., Hargreaves Z., Ho C.-S., Montoya M., Sánchez-Cordón P.J., Taylor G. (2020). A Pool of Eight Virally Vectored African Swine Fever Antigens Protect Pigs against Fatal Disease. Vaccines.

[20] Zajac M.D., Trujillo J.D., Yao J., Kumar R., Sangewar N., Lokhandwala S., Sang H., Mallen K., McCall J., Burton L. (2023). Immunization of Pigs with Replication-Incompetent Adenovirus-Vectored African Swine Fever Virus Multi-Antigens Induced Humoral Immune Responses but No Protection Following Contact Challenge. Front. Vet. Sci..

[21] Portugal R., Goldswain H., Moore R., Tully M., Harris K., Corla A., Flannery J., Dixon L.K., Netherton C.L. (2024). Six Adenoviral Vectored African Swine Fever Virus Genes Protect against Fatal Disease Caused by Genotype I Challenge. J. Virol..

[22] Fulber J.P.C., Kamen A.A. (2022). Development and Scalable Production of Newcastle Disease Virus-Vectored Vaccines for Human and Veterinary Use. Viruses.

[23] Yang H., Tian J., Zhao J., Zhao Y., Zhang G. (2024). The Application of Newcastle Disease Virus (NDV): Vaccine Vectors and Tumor Therapy. Viruses.

[24] García-Sastre A. (2022). Mucosal Delivery of RNA Vaccines by Newcastle Disease Virus Vectors. Curr. Res. Immunol..

[25] Hu Z., Ni J., Cao Y., Liu X. (2020). Newcastle Disease Virus as a Vaccine Vector for 20 Years: A Focus on Maternally Derived Antibody Interference. Vaccines.

[26] Kumar R., Kumar V., Kekungu P., Barman N.N., Kumar S. (2019). Evaluation of Surface Glycoproteins of Classical Swine Fever Virus as Immunogens and Reagents for Serological Diagnosis of Infections in Pigs: A Recombinant Newcastle Disease Virus Approach. Arch. Virol..

[27] Zhang H., Nan F., Li Z., Zhao G., Xie C., Ha Z., Zhang J., Han J., Xiao P., Zhuang X. (2019). Construction and Immunological Evaluation of Recombinant Newcastle Disease Virus Vaccines Expressing Highly Pathogenic Porcine Reproductive and Respiratory Syndrome Virus GP3/GP5 Proteins in Pigs. Vet. Microbiol..

[28] Zhang Y., Ke J., Zhang J., Yang J., Yue H., Zhou X., Qi Y., Zhu R., Miao F., Li Q. (2021). African Swine Fever Virus Bearing an I226R Gene Deletion Elicits Robust Immunity in Pigs to African Swine Fever. J. Virol..

[29] Zhang Y., Ke J., Zhang J., Yue H., Chen T., Li Q., Zhou X., Qi Y., Zhu R., Wang S. (2021). I267L Is Neither the Virulence- Nor the Replication-Related Gene of African Swine Fever Virus and Its Deletant Is an Ideal Fluorescent-Tagged Virulence Strain. Viruses.

[30] Huang Z., Krishnamurthy S., Panda A., Samal S.K. (2001). High-level Expression of a Foreign Gene from the Most 3′-proximal Locus of a Recombinant Newcastle Disease Virus. J. Gen. Virol..

[31] Ge J., Deng G., Wen Z., Tian G., Wang Y., Shi J., Wang X., Li Y., Hu S., Jiang Y. (2007). Newcastle Disease Virus-Based Live Attenuated Vaccine Completely Protects Chickens and Mice from Lethal Challenge of Homologous and Heterologous H5N1 Avian Influenza Viruses. J. Virol..

[32] Choi K.-S. (2017). Newcastle Disease Virus Vectored Vaccines as Bivalent or Antigen Delivery Vaccines. Clin. Exp. Vaccine Res..

[33] Lee J., Kim D., Noh J., Youk S., Jeong J., Lee J., Park S.-Y., Choi I., Lee S.-W., Song C. (2022). Live Recombinant NDV-Vectored H5 Vaccine Protects Chickens and Domestic Ducks from Lethal Infection of the Highly Pathogenic H5N6 Avian Influenza Virus. Front. Vet. Sci..

[34] Canter J.A., Aponte T., Ramirez-Medina E., Pruitt S., Gladue D.P., Borca M.V., Zhu J.J. (2022). Serum Neutralizing and Enhancing Effects on African Swine Fever Virus Infectivity in Adherent Pig PBMC. Viruses.

[35] Cadenas-Fernández E., Sánchez-Vizcaíno J.M., Kosowska A., Rivera B., Mayoral-Alegre F., Rodríguez-Bertos A., Yao J., Bray J., Lokhandwala S., Mwangi W. (2020). Adenovirus-Vectored African Swine Fever Virus Antigens Cocktail Is Not Protective against Virulent Arm07 Isolate in Eurasian Wild Boar. Pathogens.

[36] Neilan J.G., Zsak L., Lu Z., Burrage T.G., Kutish G.F., Rock D.L. (2004). Neutralizing Antibodies to African Swine Fever Virus Proteins P30, P54, and P72 Are Not Sufficient for Antibody-Mediated Protection. Virology.

[37] Lokhandwala S., Waghela S.D., Bray J., Sangewar N., Charendoff C., Martin C.L., Hassan W.S., Koynarski T., Gabbert L., Burrage T.G. (2017). Adenovirus-Vectored Novel African Swine Fever Virus Antigens Elicit Robust Immune Responses in Swine. PLoS ONE.

[38] Lopera-Madrid J., Osorio J.E., He Y., Xiang Z., Adams L.G., Laughlin R.C., Mwangi W., Subramanya S., Neilan J., Brake D. (2017). Safety and Immunogenicity of Mammalian Cell Derived and Modified Vaccinia Ankara Vectored African Swine Fever Subunit Antigens in Swine. Vet. Immunol. Immunopathol..

[39] Netherton C.L., Goatley L.C., Reis A.L., Portugal R., Nash R.H., Morgan S.B., Gault L., Nieto R., Norlin V., Gallardo C. (2019). Identification and Immunogenicity of African Swine Fever Virus Antigens. Front. Immunol..

[40] Bosch-Camós L., López E., Navas M.J., Pina-Pedrero S., Accensi F., Correa-Fiz F., Park C., Carrascal M., Domínguez J., Salas M.L. (2021). Identification of Promiscuous African Swine Fever Virus T-Cell Determinants Using a Multiple Technical Approach. Vaccines.

[41] Xu Z., Hu Y., Li J., Wang A., Meng X., Chen L., Wei J., Tong W., Kong N., Yu L. (2023). Screening and Identification of the Dominant Antigens of the African Swine Fever Virus. Front. Vet. Sci..

[42] Kollnberger S.D., Gutierrez-Castañeda B., Foster-Cuevas M., Corteyn A., Parkhouse R.M.E. (2002). Identification of the Principal Serological Immunodeterminants of African Swine Fever Virus by Screening a Virus cDNA Library with Antibody. J. Gen. Virol..

[43] Luong H.Q., Lai H.T., Do L.D., Ha B.X., Nguyen G.V., Vu H.L. (2022). Differential Antibody Responses in Sows and Finishing Pigs Naturally Infected with African Swine Fever Virus under Field Conditions. Virus Res..

[44] Borca M.V., Irusta P., Carrillo C., Afonso C.L., Burrage T., Rock D.L. (1994). African Swine Fever Virus Structural Protein P72 Contains a Conformational Neutralizing Epitope. Virology.

[45] Lokhandwala S., Waghela S.D., Bray J., Martin C.L., Sangewar N., Charendoff C., Shetti R., Ashley C., Chen C.-H., Berghman L.R. (2016). Induction of Robust Immune Responses in Swine by Using a Cocktail of Adenovirus-Vectored African Swine Fever Virus Antigens. Clin. Vaccine Immunol..

[46] Liu W., Li H., Liu B., Lv T., Yang C., Chen S., Feng L., Lai L., Duan Z., Chen X. (2023). A New Vaccination Regimen Using Adenovirus-Vectored Vaccine Confers Effective Protection against African Swine Fever Virus in Swine. Emerg. Microbes Infect..

[47] Blome S., Gabriel C., Beer M. (2014). Modern Adjuvants Do Not Enhance the Efficacy of an Inactivated African Swine Fever Virus Vaccine Preparation. Vaccine.

[48] Escribano J.M., Galindo I., Alonso C. (2013). Antibody-Mediated Neutralization of African Swine Fever Virus: Myths and Facts. Virus Res..

[49] Onisk D.V., Borca M.V., Kutish S., Kramer E., Irusta P., Rock D.L. (1994). Passively Transferred African Swine Fever Virus Antibodies Protect Swine against Lethal Infection. Virology.

[50] Silva E.B., Krug P.W., Ramirez-Medina E., Valladares A., Rai A., Espinoza N., Gladue D.P., Borca M.V. (2022). The Presence of Virus Neutralizing Antibodies Is Highly Associated with Protection against Virulent Challenge in Domestic Pigs Immunized with ASFV Live Attenuated Vaccine Candidates. Pathogens.

[51] Goatley L.C., Nash R.H., Andrews C., Hargreaves Z., Tng P., Reis A.L., Graham S.P., Netherton C.L. (2022). Cellular and Humoral Immune Responses after Immunisation with Low Virulent African Swine Fever Virus in the Large White Inbred Babraham Line and Outbred Domestic Pigs. Viruses.

[52] Luong H.Q., Lai H.T.L., Truong L.Q., Nguyen T.N., Vu H.D., Nguyen H.T., Nguyen L.T., Pham T.H., McVey D.S., Vu H.L.X. (2023). Comparative Analysis of Swine Antibody Responses Following Vaccination with Live-Attenuated and Killed African Swine Fever Virus Vaccines. Vaccines.

[53] Oura C.A.L., Denyer M.S., Takamatsu H., Parkhouse R.M.E. (2005). In Vivo Depletion of CD8+ T Lymphocytes Abrogates Protective Immunity to African Swine Fever Virus. J. Gen. Virol..

[54] Takamatsu H.-H., Denyer M.S., Lacasta A., Stirling C.M.A., Argilaguet J.M., Netherton C.L., Oura C.A.L., Martins C., Rodríguez F. (2013). Cellular Immunity in ASFV Responses. Virus Res..

[55] Zajac M.D., Sangewar N., Lokhandwala S., Bray J., Sang H., McCall J., Bishop R.P., Waghela S.D., Kumar R., Kim T. (2022). Adenovirus-Vectored African Swine Fever Virus Pp220 Induces Robust Antibody, IFN-γ, and CTL Responses in Pigs. Front. Vet. Sci..

[56] King K., Chapman D., Argilaguet J.M., Fishbourne E., Hutet E., Cariolet R., Hutchings G., Oura C.A.L., Netherton C.L., Moffat K. (2011). Protection of European Domestic Pigs from Virulent African Isolates of African Swine Fever Virus by Experimental Immunisation. Vaccine.

[57] Chen T.-Y., Ho Y.-J., Ko F.-Y., Wu P.-Y., Chang C.-J., Ho S.-Y. (2024). Multi-Epitope Vaccine Design of African Swine Fever Virus Considering T Cell and B Cell Immunogenicity. AMB Express.

[58] Venkateswaran D., Prakash A., Nguyen Q.A., Salman M., Suntisukwattana R., Atthaapa W., Tantituvanont A., Lin H., Songkasupa T., Nilubol D. (2024). Comprehensive Characterization of the Genetic Landscape of African Swine Fever Virus: Insights into Infection Dynamics, Immunomodulation, Virulence and Genes with Unknown Function. Animals.

[59] Wang N., Huang P., Zhang J., Lin M., Lai X., Chen J., Pan C. (2024). Advancement in the Development of Gene/Protein-Based Vaccines against African Swine Fever Virus. Curr. Res. Microb. Sci..

[60] Wang S., Zhang J., Zhang Y., Yang J., Wang L., Qi Y., Han X., Zhou X., Miao F., Chen T. (2021). Cytokine Storm in Domestic Pigs Induced by Infection of Virulent African Swine Fever Virus. Front. Vet. Sci..

[61] Zuo X., Peng G., Zhao J., Zhao Q., Zhu Y., Xu Y., Xu L., Li F., Xia Y., Liu Y. (2024). Infection of Domestic Pigs with a Genotype II Potent Strain of ASFV Causes Cytokine Storm and Lymphocyte Mass Reduction. Front. Immunol..

[62] Jiao P., Wang S., Fan W., Zhang H., Yin H., Shang Y., Zhu H., Liu W., Hu R., Sun L. (2023). Recombinant Porcine Interferon Cocktail Delays the Onset and Lessens the Severity of African Swine Fever. Antivir. Res..

[63] Jackman J.A., Hakobyan A., Grigoryan R., Izmailyan R., Elrod C.C., Zakaryan H. (2024). Antiviral Screening of Natural, Anti-Inflammatory Compound Library against African Swine Fever Virus. Virol. J..

