# Supplementary material for: Newcastle Disease Virus-Vectored African Swine Fever Virus Antigen Cocktail Delays the Onset of ASFV-SY18 but Is Not Protective
Source: Microorganisms. 2024 Dec 13;12(12):2590. doi: 10.3390/microorganisms12122590 (PMC11679766; doi:10.3390/microorganisms12122590)
Supplement: Supplementary file 1 [file microorganisms-12-02590-s001.zip › microorganisms-3325574-supplementary.pdf]

Table S1. Sizes of the constructed ASFV genes and expressed proteins

	Gene Name	Gene Size (bp)	Fragment Size by RT-PCR (bp)	Encoded Protein	Theoretical protein size (kDa)
1	A104R	315	502	pA104R	11.6
2	A118R	183	370	pA118R	7.0
3	A137R	414	601	p11.5/pA137R	16.1
4	A151R	456	643	pA151R	17.8
5	A179L	540	727	pA179L	21.1
6	A224L	675	862	pA224L	26.7
7	A238L	681	868	pA238L	26.2
8	A240L	711	898	pA240L	27.6
9	A276L	831	1018	pA276L	31.7
10	B66L	201	388	pB66L	7.6
11	B117L	348	535	pB117L	13.1
12	B119L	360	547	pB119L	14.4
13	B385R	1158	1345	pB385R	45.3
14	B438L	1317	1504	p49/pB438L	49.4
15	C62L	189	376	pC62L	7.2
16	C122R	318	505	pC122R	11.8
17	C257L	774	961	pC257L	29.7
18	C475L	1428	1615	pC475L	54.8
19	CP80R	243	430	pCP80R	9.0
20	CP123L	369	556	pCP123L	13.9
21	CP204L	385	572	p30/pCP204L	22.4
22	CP312R	924	1111	pCP312R	22.4
23	CP530R	1593	1780	pp62/pCP530R	60.5
24	D117L	354	541	p17/pD117L	13.1
25	D129L	390	577	pD129L	14.6
26	D250R	753	940	pD250R	29.8
27	D339L	1020	1207	pD339L	38.8
28	D345L	1038	1225	pD345L	39.4
29	DP60R	180	367	pDP60R	6.6
30	DP71L	213	400	pDP71L	8.4
31	DP96R	291	478	pDP96R	10.7
32	DP148R	447	634	pDP148R	17.2
33	DP238L	717	904	pDP238L	27.1
34	E66L	153	340	pE66L	6.3
35	E111L	336	523	pE111L	12.9
36	E120R	369	556	pE120R	13.9
37	E146L	441	628	pE146L	16.1
38	E165R	498	685	pE165R	18.3
39	E183L	555	742	p54/pE183L	20.0
40	E184L	555	742	pE184L	21.8
41	E199L	600	787	pE199L	22.0
42	E248R	747	934	pE248R	27.4
43	E301R	906	1093	pE301R	35.2
44	E423R	1272	1459	pE423R	48.1
45	EP84R	255	442	pEP84R	9.0
46	EP152R	459	646	pEP152R	17.8
47	EP424R	1257	1444	pEP424R	48.8
48	F165R	498	685	pF165R	19.1
49	F317L	954	1141	pF317L	36.6
50	F334L	1005	1192	pF334L	39.8
51	H108R	327	514	pH108R	12.6

	Gene Name	Gene Size (bp)	Fragment Size by RT-PCR (bp)	Encoded Protein	Theoretical protein size (kDa)
52	H124R	375	562	pH124R	14.8
53	H171R	516	703	pH171R	20.0
54	H240R	726	913	pH240R	27.7
55	H359L	1080	1267	pH359L	41.4
56	I73R	219	406	pI73R	8.4
57	I177L	534	721	pI177L	20.4
58	I226R	681	868	pI226R	27.0
59	I329L	990	1177	pI329L	38.5
60	K78R	237	424	pK78R	8.4
61	K145R	438	625	pK145R	17.2
62	K196R	591	778	pK196R	22.4
63	K205R	618	805	pK205R	23.7
64	KP177R	534	721	pKP177R	20.1
65	MGF505-5R	1497	1684	pMGF505-5R	58.6
66	MGF505-7R	1584	1771	pMGF505-7R	61.7
67	MGF110-5L-6L	618	805	pMGF110-5L-6L	23.8
68	MGF110-12L	360	547	pMGF110-12L	14.1
69	P14	462	649	P14	17.7
70	R298L	897	1084	pR298L	35.1
71	S273R	822	1009	pS273R	31.6
72	O61R	186	373	p12/pO61R	6.7
73	B646L	1971	2158	p72/pB646L	73.2
74	B602L	1593	1780	pB602L	61.3
75	F778R	2337	2524	pF778R	87.4
76	C717R	2154	2341	pC717R	83.7
